# Dissecting Outcomes: Should Cytoreductive Nephrectomy Be Performed for Patients With Metastatic Renal Cell Carcinoma With Sarcomatoid Dedifferentiation?

**DOI:** 10.3389/fonc.2020.627025

**Published:** 2021-02-10

**Authors:** Jacob J. Adashek, Yumeng Zhang, William Paul Skelton, Alyssa Bilotta, Jad Chahoud, Logan Zemp, Jiannong Li, Jasreman Dhillon, Brandon Manley, Philippe E. Spiess

**Affiliations:** ^1^Department of Internal Medicine, H. Lee Moffitt Cancer Center & Research Institute, University of South Florida, Tampa, FL, United States; ^2^Division of Medical Oncology, H. Lee Moffitt Cancer Center & Research Institute, University of South Florida, Tampa, FL, United States; ^3^Morsani College of Medicine, University of South Florida, Tampa, FL, United States; ^4^Department of Genitourinary Oncology, H. Lee Moffitt Cancer Center & Research Institute, Tampa, FL, United States; ^5^Department of Biostatistics and Bioinformatics, H. Lee Moffitt Cancer Center & Research Institute, Tampa, FL, United States; ^6^Department of Pathology, H. Lee Moffitt Cancer Center & Research Institute, Tampa, FL, United States

**Keywords:** renal cell cancer, sarcomatoid dedifferentiation, cytoreductive nephrectomy, kidney cancer, systemic therapy

## Abstract

**Background:**

It is highly contested whether cytoreductive nephrectomy for treating advanced renal cell carcinoma (RCC) with sarcomatoid features (sRCC) benefits overall survival. Patients with sRCC are known to have a poor prognosis, and these tumors have a more aggressive biology than those without sarcomatoid features.

**Methods:**

Patients with clear cell RCC or non–clear cell RCC underwent cytoreductive nephrectomy in efforts to improve overall survival (OS). Patients were stratified by presence or absence of histologic sarcomatoid features within tumor samples.

**Results:**

Of 167 patients who underwent cytoreductive nephrectomy, 127 had clear cell RCC, of whom 14 had sarcomatoid features, and 40 had non–clear cell RCC, of whom 13 had sarcomatoid features. Median age of the cohort was 62 years (range, 56.5–69 years). The cohort included 119 male (71.3%) and 48 (28.7%) female patients. Among all patients with advanced RCC, having sRCC had a significantly worse OS after cytoreductive nephrectomy (30 vs 8 months; hazard ratio [HR], 2.88; *P* <0.0001). Additionally, favorable-risk patients had significantly longer OS compared to intermediate- or poor-risk patients (56 vs 30 vs 10 months; HR, 0.21; *P* =0.00016). For patients with clear cell RCC, having sRCC conferred a significantly poorer survival (30 vs 9 months; HR, 2.82; *P*=0.0035). Patients with non–clear cell sRCC also had significantly worse outcomes compared to patients whose tumors did not have sarcomatoid features (30 vs 6.5 months; HR, 3; *P* =0.009). When patients with sRCC were stratified by whether there was >10% or ≤10% sarcomatoid features present within the sample, there was no significant difference in OS (8 vs 8.5 months; *P* =0.32).

**Conclusions:**

Sarcomatoid features within tumor histology confer significantly poor prognosis. Patients with sRCC, regardless of clear cell vs non–clear cell histology, have significantly shorter OS. Even among patients with 10% or less sarcomatoid features, there was no OS benefit to cytoreductive nephrectomy. Based on our findings, there appears to be a limited to no role of cytoreductive nephrectomy if sRCC is identified on pretreatment biopsy. The role of radiomics and pre-operative biopsies may confer significant benefit in this patient population.

## Introduction

Renal cell carcinoma (RCC) is the eighth most common malignancy in the United States in 2020 (with an estimated 73,750 cases) and accounted for the eleventh leading cause of death (with an estimated 14,830 deaths) (1, 2). There are many subtypes of RCC, the most common being clear cell RCC (approximately 70% of cases), with other subtypes including papillary, chromophobe, medullary, collecting duct, carcinoma associated with neuroblastoma, mucinous tubular and spindle cell carcinoma, and unclassified RCC (3). Most of these subtypes are rare, with the majority of the non–clear cell subtypes being papillary and chromophobe (3, 4).

Sarcomatoid transformation is characterized by a dedifferentiation process during which the malignant epithelial cells transform into malignant spindle-shaped cells. Sarcomatoid renal cell carcinoma (sRCC) is not recognized as a distinct entity and can occur in any subtype at variable proportions (3, 5). Sarcomatoid dedifferentiation occurs in only ~5% of RCCs; however, it can occur in up to 20% of advanced disease (6). It is associated with a more aggressive phenotype and confers a poor prognosis. Historically, the median overall survival (OS) for patients with sRCC is reported to be 4 to 9 months compared to 17 to 22 months for patients with nonsarcomatoid RCC (6, 7).

The pathogenesis that causes sarcomatoid dedifferentiation is poorly understood. Recent proteomic and genomic studies favor the theory of a common cell of origin, in which sarcomatoid cells arise from background carcinomatous cells by acquiring driver mutations (8–10). Sarcomatoid cells in RCC can also engage in epithelial-mesenchymal transition (EMT) (11). Patients with clear cell RCC have superior survival outcomes compared to patients with non–clear cell RCC, but this has not been demonstrated among the specific population of patients with sarcomatoid clear cell RCC compared to those with sarcomatoid non–clear cell RCC (12).

In large retrospective studies, cytoreductive nephrectomy prior to systemic therapy has been shown to improve OS for patients with metastatic RCC as compared to systemic therapies alone in the targeted therapy era (13, 14). However, sRCC is poorly represented in these studies because of its rarity. The role of CN is shrinking and the only subgroup that remains in question are those with IMDC 1, or potentially, subgroups (e.g sarcomatoid dedifferentiation) (15). The recent Clinical Trial to Assess the Importance of Nephrectomy (CARMENA) and Immediate Surgery or Surgery After Sunitinib Malate in Treating Patients With Metastatic Kidney Cancer (SURTIME) randomized clinical trials questioned the role of upfront cytoreductive nephrectomy for certain patients with metastatic RCC who are treated with tyrosine kinase inhibitor (TKI) therapy (16, 17). Unfortunately, both trials did not explore the outcomes of patients with sRCC. As compared to nonsarcomatoid RCCs, sRCCs carry more aggressive phenotypes and higher propensity for metastases, and patients with sRCC have suboptimal outcomes despite immunotherapies or targeted therapies (18). Therefore, the role of cytoreductive nephrectomy needs to be assessed separately, especially with emerging immunotherapies.

Systemic therapies are dependent on histology as well as risk stratification, but regimens are largely based on immunotherapy and TKIs. For patients with intermediate- or poor-risk disease, therapeutic options include ipilimumab + nivolumab, axitinib + pembrolizumab, and axitinib + avelumab, in addition to TKI therapy. Of note, these regimens were all FDA approved for clear cell histology (19–21). A subgroup analysis of the patients with clear cell sRCC within the ipilimumab + nivolumab trial were found to benefit from this regimen with an objective response rate of ~61%, a ~19% complete response rate, and an overall survival that was not reached in the trial (22).

The objective of this study was to evaluate the impact of cytoreductive nephrectomy for patients with metastatic sRCC and their survival outcomes.

## Methods

### Patients and Study Design

Under Institutional Review Board approval (MCC 15666), we retrospectively reviewed data from patients diagnosed with metastatic RCC who underwent cytoreductive nephrectomy at H. Lee Moffitt Cancer Center and Research Institute between 2008 and 2019.

The baseline clinical and treatment data that were extracted from electronic medical records included age, gender, race, vital status, date of diagnosis, date of last visit/death, Eastern Cooperative Oncology Group (ECOG) status, IMDC criteria (23), presence of lymphadenopathy, and metastatic sites. A genitourinary-specific pathologist reviewed the surgical specimens and determined the tumor histology, size, and grade. The pathologist also verified the presence of sarcomatoid features and estimated the percentage of sarcomatoid features in the specimen.

### Study Statistical Analyses

There were a total of 167 patients in this retrospective study cohort. In order to evaluate the association between patients with metastatic sRCC and their OS, patients were stratified by the presence of any sarcomatoid dedifferentiation, defined as 1% or greater, with sarcomatoid features defined as spindling tumor cells which are pleomorphic (range, 3%–100%) and by the histology of the cancer (clear cell vs non–clear cell). Baseline characteristics were summarized using descriptive statistics, including median and interquartile range for continuous variables and proportions and frequencies for categorical variables. Kruskal-Wallis tests for continuous variables and chi-squared tests or Fisher exact tests for categorical variables were conducted to compare their difference.

For survival analyses, survival time for all patients was defined as the time of the cytoreductive nephrectomy to the time of death from any cause or censorship at the last follow-up date. The Kaplan-Meier method was used for OS analyses, and log-rank tests were adopted to compare survival differences between two groups. To evaluate the association of OS with individual clinical and pathological features, we first conducted univariate Cox proportional hazards models, and then, for those predictors that have a potential association with OS (*P* < .25), the multivariate Cox proportional hazards models were performed to further evaluate their association with OS. All statistical analyses were performed using SAS (version 9.4, SAS Institute Inc., Cary, NC) and the R 3.6.0 software (https://www.R-project.org).

## Results

### Demographics and Clinical Characteristics

A total of 167 patients with RCC underwent cytoreductive nephrectomy over an 11-year span at H. Lee Moffitt Cancer Center and Research Institute. Among them, 16% of patients (n = 27/167) had tumors with sarcomatoid dedifferentiation noted in the final pathology reports. **Table 1** displays the patients’ demographic and clinical characteristics.

**Table 1:** Demographic and clinical characteristics of 167 patients with metastatic renal cell carcinoma who underwent cytoreductive nephrectomy.

Variable	Whole Cohort		sRCC		non-sRCC		p value
	Count	Percentage (%)	Count	Percentage (%)	Count	Percentage (%)	
**No**.	167		27	16.2	140	83.8	
**median age at presentation**	62	95% CI 57–69	63	95% CI 57–67	62	95% CI 57–69	0.728
**Sex**							0.13
female	48	28.7	4	14.8	44	31.4	
male	119	71.3	23	85.2	96	68.6	
**Race**							0.387
White	146	87.4	24	88.9	122	87.1	
Black	4	2.4	2	7.41	2	1.43	
Hispanic	4	2.4	0	0	4	2.86	
Asian	3	1.8	0	0	3	2.14	
Other	10	6	1	3.7	9	6.43	
**Histology**							0.003
Non clear cell	40	24	13	48.1	27	19.3	
Clear cell	127	76	14	51.9	113	80.7	
**Grade**							<0.001
<=2	18	10.8	0	0	18	12.9	
3	101	6.8	3	11.1	98	70.5	
4	47	28.3	24	88.9	23	16.5	
**Number of metastases**							0.005
1	93	55.7	8	29.6	85	60.7	
>=2	84	44.3	19	70.4	55	23.6	
**IDMC risk**							0.081
poor	36	21.6	9	33.3	27	19.3	
intermediate	117	70.1	18	66.7	99	70.7	
Good	14	8.38	0	0	14	10	
**ECOG**							1
0**–**1	132	95	21	95.5	111	94.9	
>1	7	5.04	1	4.55	6	11.5	
**Serum calcium (g/L)**							1
<10.2	92	78	13	76.5	79	78.2	
>=10.2	26	22	4	23.5	22	21.8	
**Neutrophil**							0.184
<7	54	85.7	8	72.7	46	88.5	
>=7	9	14.3	3	27.3	6	11.5	
**Hemoglobin (g/dl)**							0.039
<= 12	84	50.3	19	70.4	65	46.4	
>12	83	49.3	8	29.6	75	53.6	
**Platelet (× 10^9/L)**							0.369
<150	10	6	0	0	10	7.14	
>=150	157	94	27	100	130	92.9	
**Lymphadenectomy**							0.019
No	87	52.1	8	29.6	79	56.4	
Yes	80	47.9	19	70.4	61	43.6	

### Survival and Prognostic Factors

**Figure 1:**
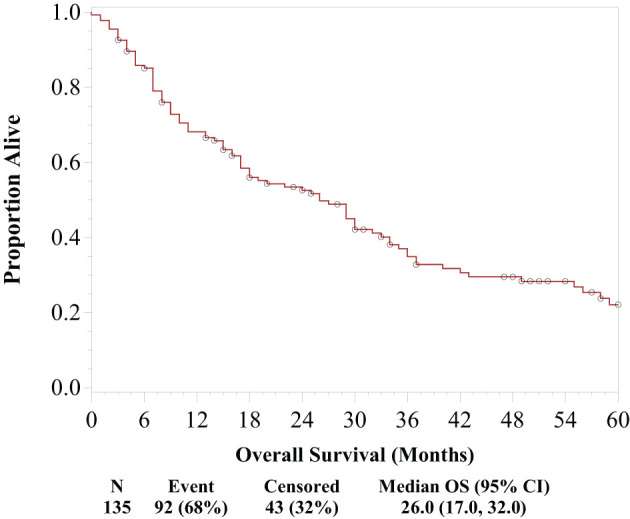
Overall survival of 167 patients with metastatic renal cell carcinoma who underwent cytoreductive surgery.

At the median follow-up time of 57 months, 68.1% (n = 92/167) of patients were deceased. **Figure 1** illustrates the survival of patients with metastatic RCC who underwent cytoreductive surgery. Median OS was 26 months (95% CI, 17–32 months). **Table 2** shows univariate and multivariable Cox proportional hazards models for the whole cohort of patients with metastatic RCC after cytoreductive surgery.

**Table 2:** Univariate and multivariate predictors of overall survival in patients with metastatic renal cell carcinoma who underwent cytoreductive nephrectomy.

Variable	Univariate Model			Multivariate Model		
	HR	95% CI	P	HR	95% CI	p value
**Median age at presentation**	1.01	0.99–1.03	0.388			
**Histology**						
non clear cell	Reference		–	Reference		–
clear cell	0.73	0.45–1.17	0.19	1.03	0.47–2.36	0.94
**Grade**						
<=2	Reference		–			
3	1.28	0.58–2.85	0.54	1.50	0.30–7.55	0.62
4	5.6	2.42–12.9	<0.001	14.77	2.35–92.74	0.004
**Sarcomatoid feature**						
Absent	Reference			Reference		
Present	2.88	1.71–4.83	<0.001	1.07	0.44–2.64	0.87
**Number of metastases**						
1	Reference		–	Reference		–
2	1.53	0.97–2.43	0.07	0.32	0.11–0.96	0.04
>=3	1.63	0.92–2.88	0.09	0.67	0.20–2.23	0.51
**IDMC risk**						
Poor	Reference		–	Not performed on multivariable analysis due to confound with other clinic features		
Intermediate	0.44	0.27–0.70	<0.001			
Good	0.21	0.08–0.54	0.001			
**ECOG**						
0**–**1	Reference		–	Reference		–
>1	2.44	0.88–6.67	0.087	5.59	1.46–21.34	0.01
**Serum calcium (g/L)**						
<10.2	Reference		–	Reference		–
>=10.2	2.34	1.34–4.07	0.003	2.24	1.05,4.78	0.04
**Neutrophil**						
<7	Reference		–			
>=7	1.42	0.55–3.65	0.47			
**Hemoglobin (g/dl)**						
<= 12	Reference		–	Reference		–
>12	0.38	0.25–0.59	<0.001	0.72	0.33–1.56	0.4
**Platelet (× 10^9^/L)**						
>=150	Reference		–	Reference		–
<150	1.68	0.84–3.37	0.144	4.85	1.32–17.87	0.02
**Lymphadenectomy**						
No	Reference		–	Reference		–
Yes	1.46	0.96–2.21	0.074	1.09	0.46–2.36	0.95

### Sarcomatoid Dedifferentiation Is a Poor Prognostic Factor

**Figure 2:**
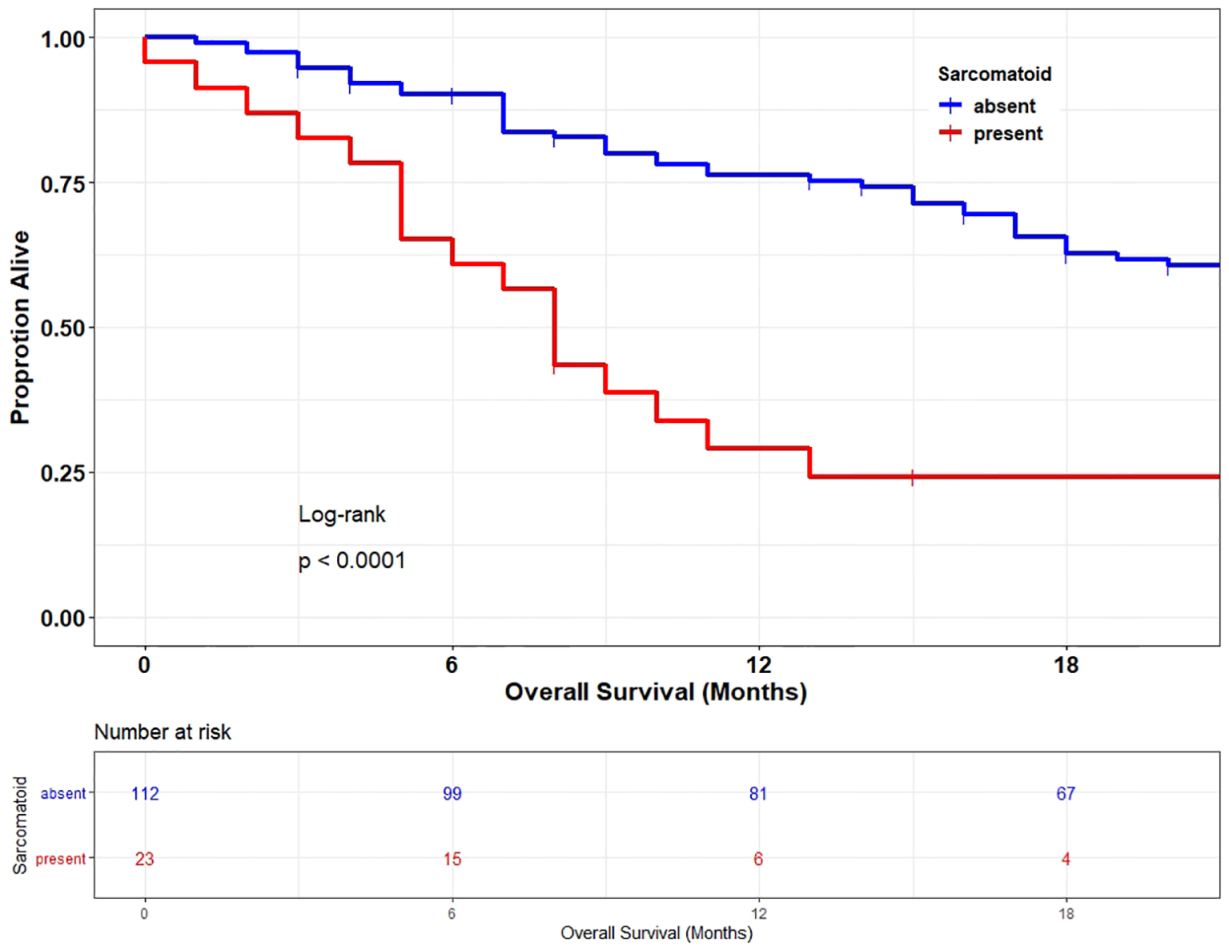
Overall survival of patients with metastatic renal cell carcinoma stratified by the presence or absence of sarcomatoid dedifferentiation.

As shown in **Figure 2**, patients with sRCC had a significantly shorter median OS compared to those with nonsarcomatoid RCC (8 months vs 30 months; HR, 2.88; *P* <0.0001). When we compared patients with RCC with and without sarcomatoid dedifferentiation, patients whose tumors had sarcomatoid dedifferentiation more frequently had histologically grade 4 disease (88.9% vs 16.5% for sRCC and nonsarcomatoid RCC, respectively; *P* <0.001) as well as polymetastatic disease (70.4% vs 39.3% for sRCC and nonsarcomatoid RCC, respectively; *P* =0.005). Interestingly, patients with sRCC were more likely to undergo lymphadenectomy (56.4% vs 29.6%; *P* =0.019), likely because of more aggressive features observed intraoperatively.

**Figure 3:**
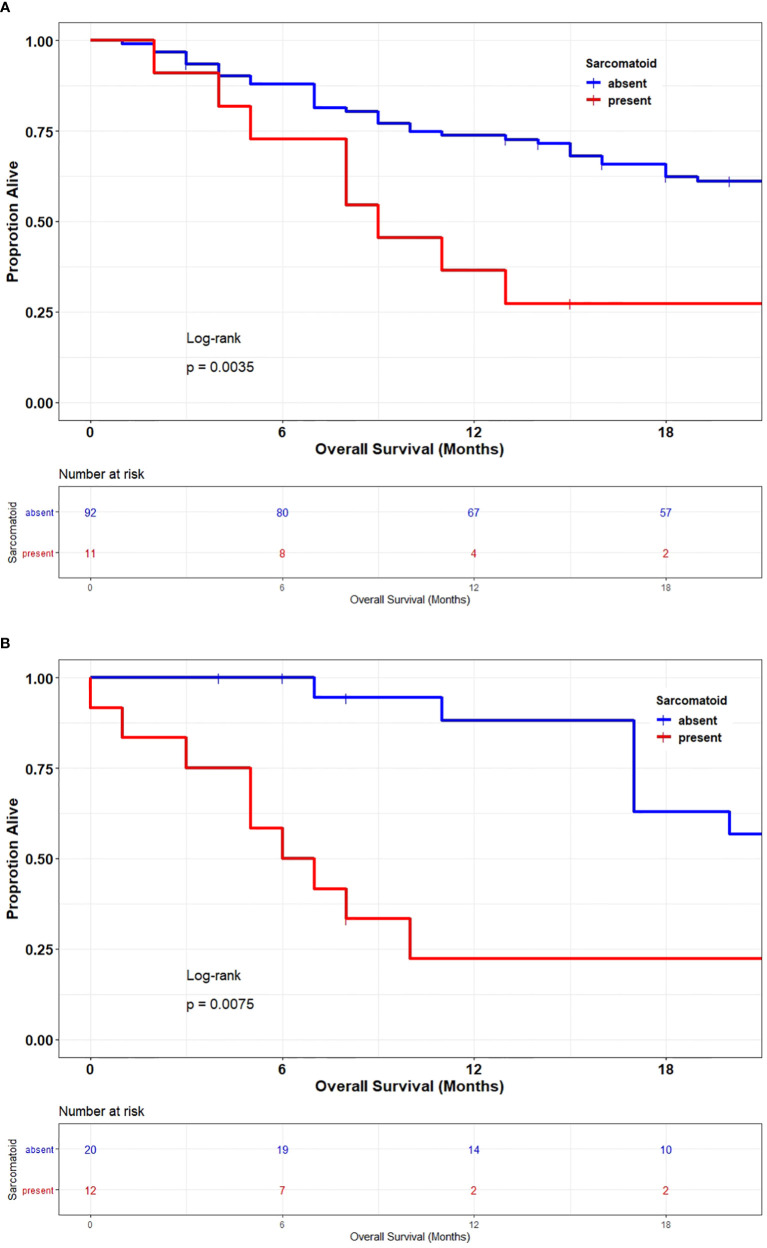
**(A)** Overall survival of patients with metastatic clear cell renal cell carcinoma stratified by the presence or absence of sarcomatoid dedifferentiation. **(B)** Overall survival of patients with non-clear cell renal cell carcinoma stratified by the presence or absence of sarcomatoid dedifferentiation.

**Figure 3** stratified patients by clear cell and non–clear cell histology. **Figure 3A** demonstrated that patients whose tumors had sarcomatoid dedifferentiation had a significantly worse 1-year survival rate after cytoreductive surgery as compared to the sarcomatoid-absent group (18% vs 90%, respectively; HR, 2.85; *P* =0.0035), and the **Figure 3B** demonstrated similar survival curves among patients whose tumors had non–clear cell histology (30 months vs. 6.5 months; HR, 3; P =0.009).

### Role of Lymphadenectomy in Cytoreductive Surgery for Metastatic RCC

We first evaluated the impact of lymphadenectomy on the OS of all patients with metastatic RCC who were undergoing cytoreductive surgery. In the univariate analyses, lymphadenectomy showed a trend of association with poor prognosis (HR, 1.46 [95% CI, 0.96–2.21]; *P* =0.074). However, after adjusting for other factors, this association was no longer significant (HR, 1.09 [95% CI, 0.46–2.36]; *P* =0.95). Interestingly, in the multivariable analyses of the group without sarcomatoid dedifferentiation, patients who had a lymphadenectomy had improved survival (HR, 0.29 [95% CI, 0.09–0.90]; *P* =0.03).

## Discussion

The role of cytoreductive nephrectomy for patients with sRCC has long been debated (24). It has been demonstrated that sarcomatoid features portend a worse prognosis regardless of clear cell or non–clear cell histology (25). The percentage involvement by sarcomatoid differentiation correlates with worse survival outcomes (26). Although the role of cytoreductive nephrectomy in this particular disease subset has continued to be debated, new evidence from a large cohort at Memorial Sloan Kettering Cancer Center found that, among patients whose tumors have sarcomatoid differentiation, a subset of patients may benefit from cytoreductive nephrectomy if they have clear cell histology, unifocal metastasis not involving the lung or liver, and node-negative disease (27). It is important to note that this is for all patients with sRCC, not only for those with metastatic cases (28–30). It has been shown that atezolizumab + bevacizumab for patients with variant histology or more than 20% sarcomatoid dedifferentiation showed an improvement in progression-free survival (median progression-free survival, 8.3 months [95% CI. 5.7–10.9]) (31).

We also have shown that patients whose diseases have sarcomatoid features who have undergone cytoreductive nephrectomy have far worse outcomes compared to patients whose diseases don’t have sarcomatoid features. This effect was extremely significant, with an 18% 1-year survival rate for patients with RCCs with sarcomatoid features after cytoreductive nephrectomy compared to 90% for patients with RCCs without sarcomatoid features. It is important to note that this pronounced difference in survival is found regardless of the exact percentage of sarcomatoid features. Therefore, if there was any clinically significant sarcomatoid aspect (>1%), there was no benefit to cytoreductive nephrectomy. Of note, over half (n = 14/27 [52%]) of our patients had less than 50% sarcomatoid features, which further supports that any sarcomatoid differentiation is associated with worse outcomes.

It is important to note that with regards to the findings that the presence of sarcomatoid features conferring a worsened survival outcome, that this was shown on univariate analysis (HR 2.88, 95% CI 1.71–4.83, p<0.001), however this was not shown on multivariate analysis (HR 1.07, 95% CI 0.44–2.64, p=0.87) with all factors (**Table 2**). Examining this data with respect to the factors that were significantly different between the cohorts of sarcomatoid and non-sarcomatoid features (histology, grade, number of metastasis, hemoglobin, and whether the patient underwent lymphadenectomy) as shown in **Table 1**, analysis confirmed these findings, however only the grade of the tumor played an important role in this outcome. When controlling for grade, analysis showed that the presence of sarcomatoid features is significantly associated with worsened overall survival (HR 2.23, 95% CI 1.19–4.06), and the median OS was 29.0 months for patients with the absence of sarcomatoid features (95% CI 17.0–34.0 months), compared to 8.0 months for patients with the presence of sarcomatoid features (95% CI 5.0–13.0 months), supporting our findings.

Univariate analyses of our findings showed that there were worse survival outcomes among patients with tumors of a higher histologic grade, hypercalcemia, and low hemoglobin, which supports the IMDC Risk Score model (it is important to note that the IMDC Risk Score was validated to determine OS among patients with metastatic RCC who underwent systemic therapy). Multivariate analyses, on the other hand, demonstrated worse survival outcomes for patients with a poor performance status as well as thrombocytopenia. This differs some from the IMDC Risk Score for RCC, as worse prognostic markers in that model include faster time to progression, poor performance status, low hemoglobin, hypercalcemia, elevated neutrophil count, and thrombocytosis.

One potential explanation for why our study found worse outcomes associated with thrombocytopenia compared to the IMDC model showing worse outcomes with thrombocytosis could be that our study examined a subset of patients who underwent cytoreductive nephrectomy. As such, patients who had thrombocytopenia and underwent a surgical procedure could have been at higher risk for postoperative complications, especially bleeding. It is important to note that, when the multivariate analyses was corrected for the potential confounding factors of the IMDC Risk Score, the thrombocytopenia became non–statistically significant (*P* =.369), although patients with platelets < 150 whose tumors had sarcomatoid features were 0% (n = 0/27) compared to 7.1% (n = 10/140) of patients with platelets > 150.

With regards to lymphadenectomy, our results showed that it was associated with a trend (albeit non–statistically significant) toward poor survival in univariate analyses. However, in multivariable analyses adjusting for other factors, this trend was not seen, likely because patients with more advanced disease (clinically regionally node-positive disease as seen pre- or intraoperatively) for whom a regional lymphadenectomy was performed, as it was deemed to be beneficial for diagnostic and/or therapeutic purposes.

In contrast, among the subset of patients with nonsarcomatoid RCC, patients who underwent lymphadenectomy were actually shown to have a survival advantage. When combining these findings with the OS of patients with sRCC vs nonsarcomatoid RCC, it is plausible that the reason that patients with nonsarcomatoid RCC gleaned a survival advantage from lymphadenectomy was that the cancer was relatively more localized at that point in time compared to with sRCC, for which, even if no clinically apparent sites of metastasis are seen, there is a high chance that micrometastatic disease beyond the regional lymph nodes has already developed, thereby limiting the benefit of cytoreductive nephrectomy in this patient population.

Clearly, these outcomes are difficult to predict in a prenephrectomy situation, as most often the tumor histology is not known prior to taking the patient to the operating room for nephrectomy. Though the majority of our study findings conferred poor prognostic outcomes similar to the IMDC model (hypercalcemia, anemia, and poor performance status) and indicated higher histologic grade unable to be assessed prior to surgical resection, the thrombocytopenia that we have seen associated with a worse survival outcome among patients with sRCC may suggest that these patients are more likely to have sRCC and thereby not benefit from cytoreductive nephrectomy. It is also important to note that regardless of if patients have sRCC or nsRCC, optimal patient selection based on their IMDC risk plays a role as to whether cytoreductive nephrectomy has a benefit in patients with synchronous metastasis (32), as well as if cytoreductive nephrectomy can be deferred in a subset of patients with newly diagnosed metastatic RCC (33). While this is applicable to metastatic RCC as a whole and not particularly of the sarcomatoid variant, it remains an important consideration. Further large-scale studies are needed to validate these findings and further elucidate other potential risk factors to create a model for identifying this subset of patients.

There is a great deal of research attempting to identify patients whose diseases may have sarcomatoid features (34). If we are able to identify these patients prenephrectomy, it would portend superior outcomes. The 2 main areas of research needed to identify these patients are preoperative biopsies and radiomics. It has been shown that, for patients with large renal masses, a biopsy from a single location is suboptimal to accurately determine histologic features. Abel et al. from the University of Wisconsin found that standard core renal biopsies are insufficient to determine sarcomatoid features and that a multiquadrant technique (obtaining core biopsy samples from at least 4 separate solid enhancing areas of tumor) increases the sensitivity of identifying samples that harbor sarcomatoid features (35). They found that using the multiquadrant biopsy technique resulted in an 86.7% chance of identifying sarcomatoid features compared to a 25% chance of identifying sarcomatoid features from a single biopsy (35). These findings suggest that using a multiquadrant biopsy technique rather than a single biopsy significantly increases physicians’ ability to determine whether tumor samples harbor sarcomatoid features and thereby provide important diagnostic and prognostic information as to the utility of a cytoreductive nephrectomy.

Radiomics is also a promising field to predict if patients may have sarcomatoid features *via* radiographic imaging. This field investigates numerous radiographic findings to incorporate into data algorithms which are not visually apparent. It has been determined that radiographic features can be used to accurately determine whether patients have sarcomatoid or nonsarcomatoid features (36). This represents a remarkable advance in the field of RCC, as, if the composition of radiographic features through radiomics can be used to accurately predict which patients have sarcomatoid features, this would have tremendous prognostic and treatment impact for all patients with RCC with sarcomatoid dedifferentiation.

It has also been demonstrated that patients with sRCC who undergo cytoreductive nephrectomy have worse survival outcomes (HR, 5.822) if they do not undergo postoperative systemic therapy (37), which is concordant with our earlier findings that sole cytoreductive nephrectomy is not sufficient in this cohort because of worse survival and also that, without treatment (despite limited options with variable efficacy), patients do worse than those who do not receive any chemotherapy at all.

The strengths of our study include our strong pathology department at a tertiary NCCN (National Comprehensive Cancer Center) referral center, as they were able to elucidate the percentage of sarcomatoid features on all the histological specimens. Another strength of the study is that we were able to examine a large sample size of this rare malignancy to provide analysis with sufficient power to make conclusions which can help in this rare patient population with limited data.

The limitations of our study are inherent to its design as a retrospective review. Additionally, it was conducted as a single-center analysis so these findings may not be representative of the country or world as a whole. Further, this is a small retrospective study during relatively long period and the systemic therapy following cytoreductive nephrectomy seems to be various. The role of cytoreductive nephrectomy may be changing in the immune checkpoint inhibitor era; however, this report lacks these patients.

## Conclusion

Cytoreductive nephrectomy has a limited role for patients with metastatic RCC. Among patients with a high kidney tumor volume, those who have good-risk features based on the IMDC Risk Score, and those who are symptomatic, cytoreductive nephrectomy may play a role in treatment, especially if pretreatment systemic therapy provides a benefit. However, among patients with extensive metastatic disease or those who are otherwise poor-risk, it does not contribute to an improvement in outcomes. In fact, it may be detrimental, especially for patients with thrombocytopenia, as not only do they not have improved outcomes with cytoreductive nephrectomy, but they have increased mortality from postoperative complications. Identifying sarcomatoid features preoperatively has been a topic of great interest, as, if physicians were able to identify these patients before the patient is taken to the OR, it would portend better outcomes. Radiomics as well as the role of preoperative multiquadrant biopsies are exciting areas of research under review to determine if these approaches can serve a benefit. Treating patients in a personalized, unique approach may be needed to further improve outcomes for patients with sRCC and utilization of genomic and transcriptomic data to further provide insight into treatment strategies (38–40). Further large-scale studies are needed to determine the optimal population of patients with sRCC who may derive an effect from cytoreductive nephrectomy.

## Data Availability Statement

The raw data supporting the conclusions of this article will be made available by the authors, without undue reservation.

## Ethics Statement

The studies involving human participants were reviewed and approved by Institutional Review Board approval (MCC 15666). The ethics committee waived the requirement of written informed consent for participation.

## Author Contributions

AB collected data for the study. JL completed statistical analysis for the study. JD completed pathologic analysis of all samples. JJA, YZ, and WS drafted the manuscript. All authors contributed to the article and approved the submitted version.

## Conflict of Interest

The authors declare that the research was conducted in the absence of any commercial or financial relationships that could be construed as a potential conflict of interest.
